# Religion would be a missing link in the case formulation of adolescents with conduct problems: an eclectic approach

**DOI:** 10.3389/fpsyt.2024.1348799

**Published:** 2024-04-22

**Authors:** Zeynep Goker

**Affiliations:** Child and Adolescent Psychiatry, Ankara City Hospital, Ankara, Türkiye

**Keywords:** Islam, conduct (behavioral) problem, religion, adolescent, prayer, body-mind, body-spirit, children

## Abstract

Psychiatry is concerned with mental health. Cognition is one of the key mental functions and manifests itself primarily as behavior. A behavior exhibited in response to a stimulus is influenced by biological (inherited), psychological (individual), and social (environmental) factors. During consolidation of an exhibited behavior, the factors affecting the individual’s cognitive structure and personality play crucial roles. Underlying factors for a problematic behavior, and their weakness/strength levels are determined via the Biopsychosocial model. Empirically effective current practices to intervene the problematic behaviors do not always result in success. One of the reasons may be other elements that were omitted during the case formulation process. This article aims to stress the idea that whatever the underlying factor of a problematic behavior is, the most crucial determinant and/or pre-emptive factor in developing or maintaining that behavior might actually be the religion as a governing and directive philosophy on how to conduct oneself. In this instance, the key is in the hands of the parents or caregivers.

## Introduction

**Figure 1:**
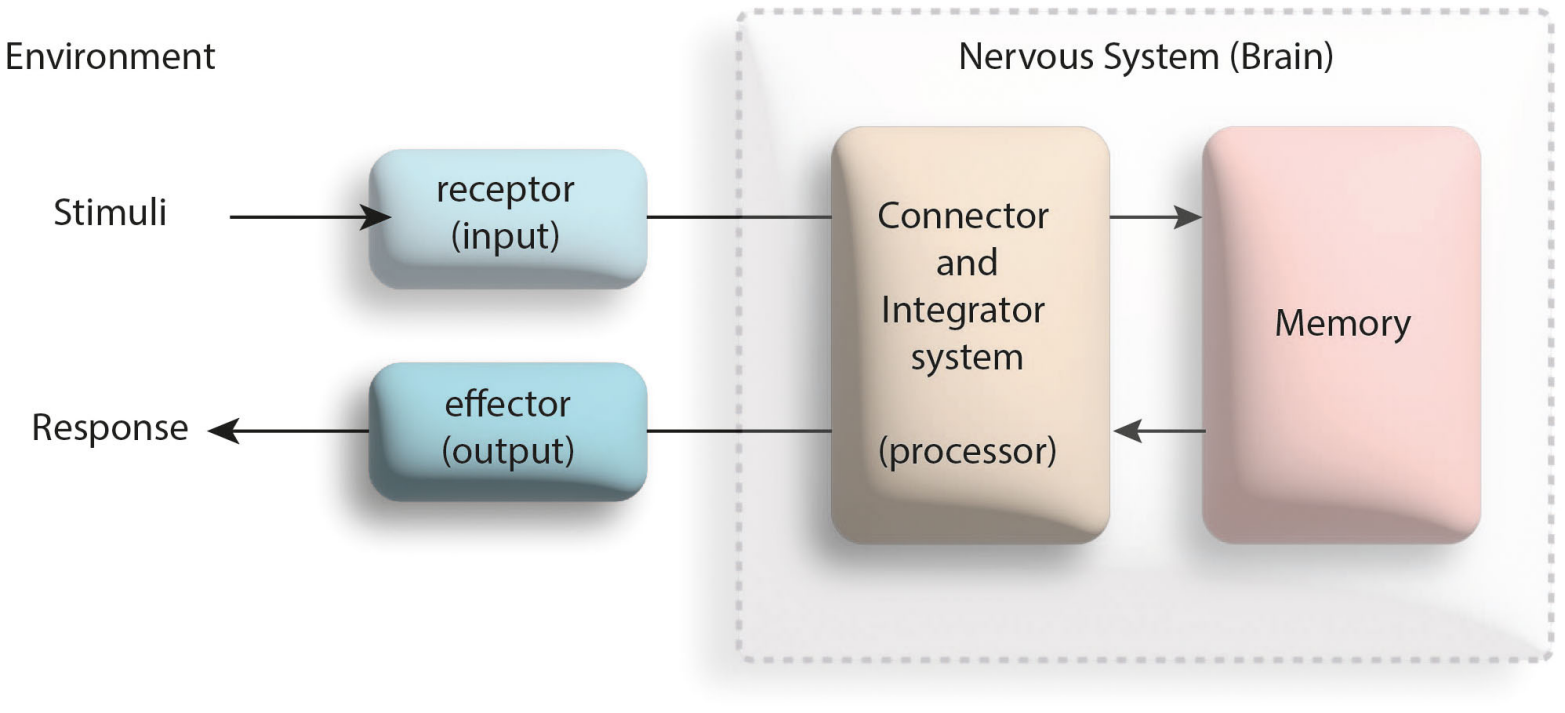
Effective factors in the process of responding to a physical or mental stimulus (modified from the reference 1).

Behavior, which is evaluated on the body-mind axis in the medical literature in general and psychiatry in particular, is, a reaction developed at the mental level to the effects caused by stimuli. Sensory or motor stimuli that evoke the bodily receptors are converted into the reaction by the cognitive structure, which is an important part of the mind. The reactions that develop with the activation of cognitive structure elements such as memory, attention, judgment, problem solving and decision making are transformed into a behavior via the relevant effector organs. In the course of processing stimuli and transforming them into a behavior, the brain is both the integrator that processes the stimuli as well as the connector between the receptor and the effector (1) (see **Figure 1**).

**Figure 2:**
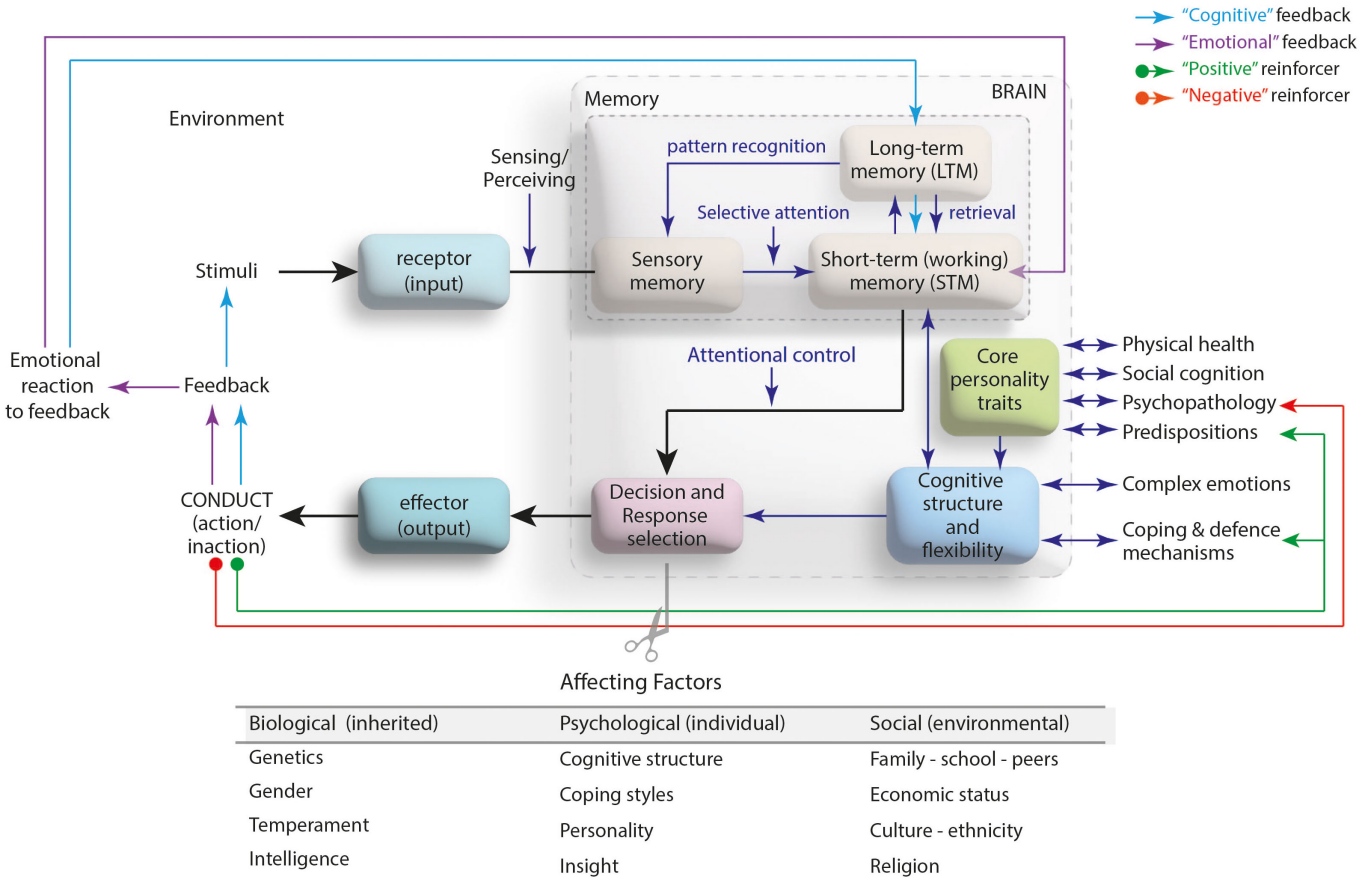
The transformation of a stimuli into a behavioral output: Mind-brain axis (modified from the references 3–8).

As an expression of the cognitive structure functioning of the human mind, behavior is under the influence of the biological (inherited), psychological (individual, acquired), and social (environmental) factors (2). The inherited factors such as genetics, the individual elements such as personality, and the environmental factors like religion all are effective in giving a wide variety of responses to the very same stimulus, which may vary from individual to individual (see **Figure 2**). In the process of the reinforcement of an exhibited behavior via the feedback, the characteristics of individual’s personality and the cognitive structure play critical roles. The personality, a part of the individual that is open to the development throughout his/her life, is formed by the combination of the individual’s temperament and character elements. During its development in psychosexual and psychosocial contexts, the personality is affected by a wide variety of elements, such as the individual’s physical health, the presence of a psychopathology, and the individual predispositions (9). The cognitive structure, on the other hand, develops in four stages according to the Piaget; the sensory-motor, the pre-operational, the concrete-operational and the formal-operational, and is mainly affected by the individual’s coping skills and defense mechanisms (3) and complex emotions (4). While a gain caused by a behavior is a positive reinforcer for the individual’s coping and defense mechanisms (5), the very same outcome can play a negative reinforcer role, for example, in the presence of a psychopathology (6) (see **Figure 2**).

In order for a behavior to be a disorder that requires medical intervention, it must meet some certain criteria in form, duration and frequency terms. Medical intervention for a disorder diagnosed based on the diagnostic systems such as the International Classification of Diseases (ICD), Diagnostic and Statistical Manual of Mental Disorders (DSM) is carried out through a case formulation. That is, possible biological, psychological and social factors underlying that disorder, the weaknesses/strengths of these factors, and the individual’s personality characteristics and cognitive structure are determined and the points to intervene are defined (7). The aim of medical intervention is to, first, do no harm (primum non nocere) (8), avoid stigmatization (10) and prevent the development of some mental disorders (11).

Medical interventions like therapeutic and pharmacological applications target the mind-brain axis (12). The goals of the treatment are to eliminate the disorder completely (cure), if this is not possible, then to prevent it from recurring (relapse), or if it is persistent, to minimize or alleviate the effects of that disorder (remission). Thus, it is aimed to increase the person’s individual and social functionality and quality of life (13). While it is possible to achieve goals in the treatment of some structural-behavioral disorders caused by biological factors, depending on the nature of the disorder, this is not always possible in some functional-behavioral disorders in which some psychological and social factors come to the fore in their etiology. Current therapeutic interventions including cognitive-behavioral therapy and pharmacological practices are not always sufficient to eradicate some problematic behaviors such as substance use disorder (14), impulse control disorder (15), impaired eating behavior (16), self-harming behavior (17) or committing crime (18). One of the reasons for this may be that the religion factor was not taken into consideration during the case formulation.

**Figure 3:**
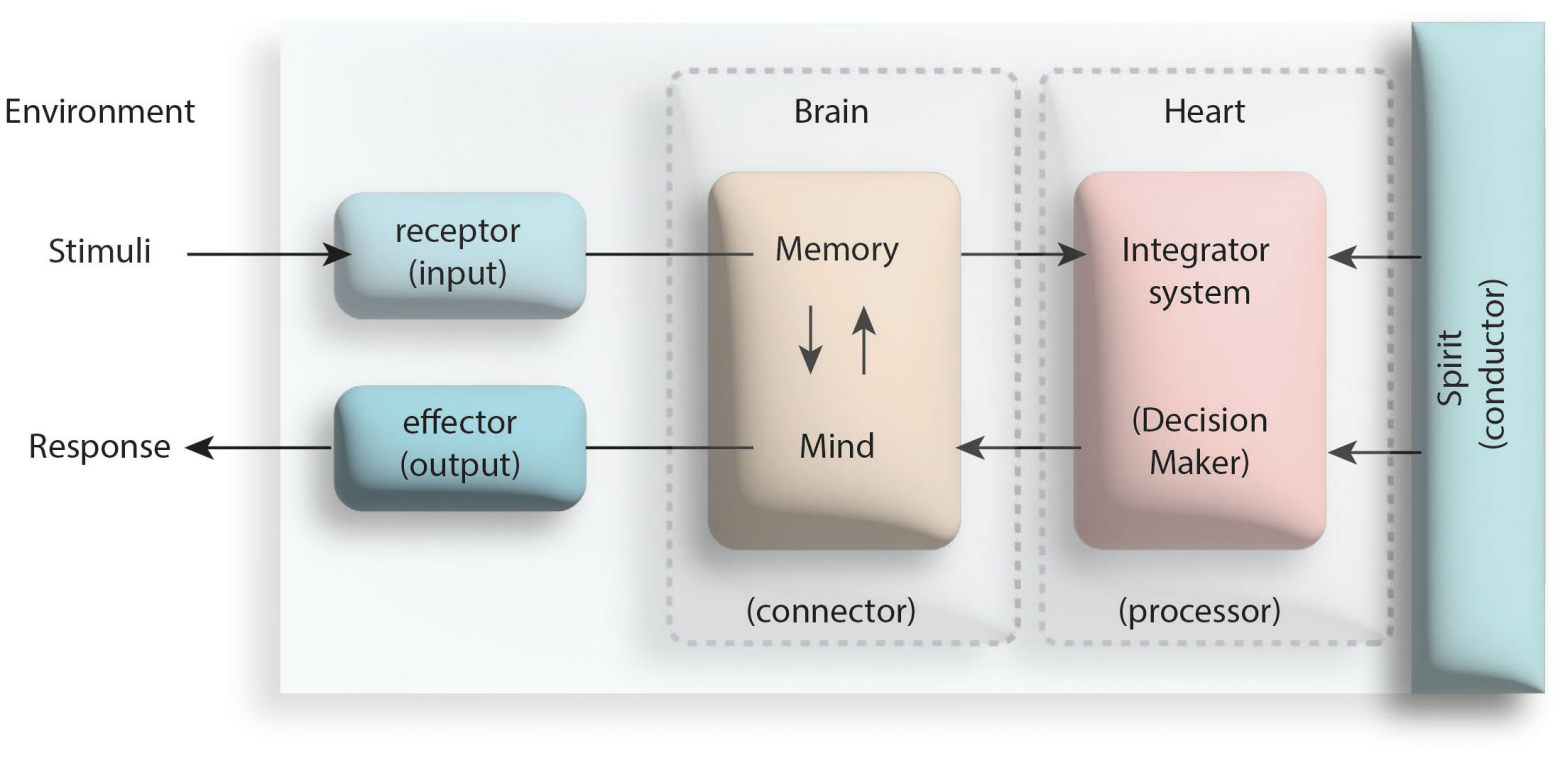
The central role of the heart (qalb) in the process of behavioral response to a stimulus (modified from the reference numbered 20).

The concept of religion, which is nourished by many sources, is a set of provisions directed to the people by a supreme creator, according to the Abrahamic religions. Based on this religious perspective, which considers humans on the body-spirit axis, behavior is a manifestation of the function of the spirit device in humans. The spirit (soul), which is the source of vitality of the body, exists to give people the awareness (divine inspiration) that they are created and that they have a creator. According to the Islamic perspective, the spirit performs this function through the heart (qalb) device, where it contacts the body (19). The heart, which is closely related to the blood-pumping organ of the human being, is the device of the person that recognizes and knows his/her creator and directs his/her body to the provisions of Him (19). In other words, a person performs a behavior in response to a stimulus he/she encounters through his/her heart; stimuli reaching the brain through receptors are transmitted from there to the heart, that is, to the center of decision making where the stimuli are processed. Here, the reactions shaped by the activation of some structural instruments belonging to the heart such as desire/inspiration, tenacity/volition and intention. The decisions made by the heart are transmitted to the relevant effector organs to be transformed into a behavior (20). In the process of converting stimuli into a behavior, the brain is only a connector/interface between the processor heart device and the receptor/effector organs (see **Figure 3**).

**Figure 4:**
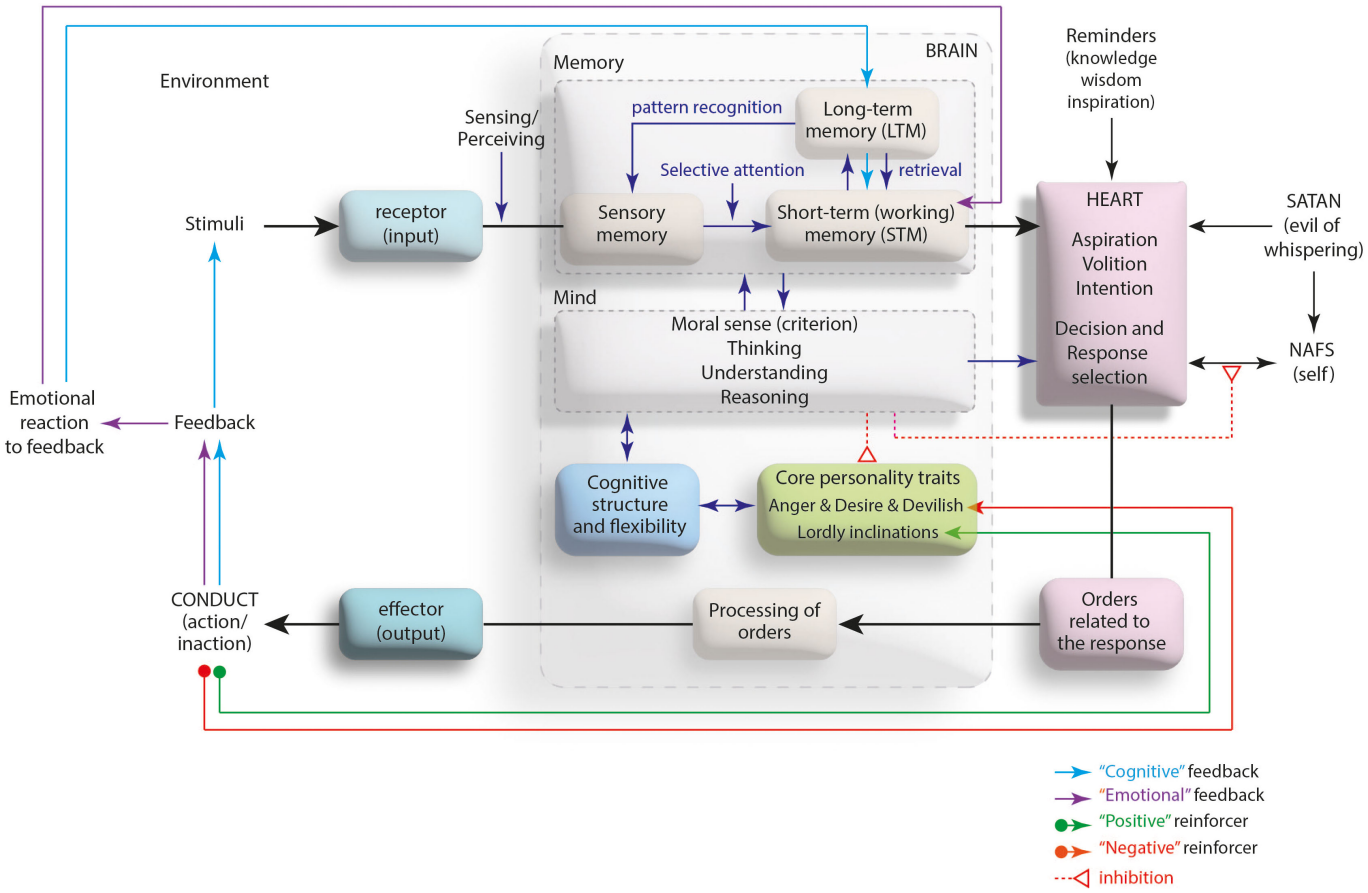
The devices playing a role in the development and reinforcement of a behavior: Mind-heart axis.

As an expression of the functioning of the spirit through the heart device, behavior is affected by some structural and environmental factors of the individual. The inherited factors such as self (nafs), the individual elements like mind (aql), and the environmental issues including the reminders (knowledge, wisdom and inspiration that the heart has acquired) and the Satan (evil of the whispering, elusive tempter), all affect the heart’s decision-making processes resulting in giving a wide variety of responses to the same stimulus, which may vary from individual to individual (21). Self (nafs) is the organ where a person’s unique impulses, instincts and tendencies (such as seeking of pleasures, power or meaning) are located. By motivating these structural elements, the self wants to make what it desires look desirable to the heart and arouse a desire in it. The mind apparatus, on the other hand, is the organ that enables a person to think and understand the surroundings with their qualities (moral sense, faculty of reasoning, criterion). The mind tries to prevent the heart from being under the influence of the self by guiding the heart to the extent of the knowledge and healthy thinking ability that has in its structure. The Satan, as the Quran (Al-Isra 17/53) stated it as a “Man’s open foe!” (22), tries to influence people’s decision-making mechanisms either directly through spectres/insinuations (waswasah) or indirectly through the self. As regards the process of the reinforcement of an exhibited behavior via the feedback is determined by the religious background of the individual’s personality. According to the Ghazali, there are four elements that make up personality; faculty of desire (to eat, drink and procreate; lusts), faculty of anger (desire to protect oneself from threats; wrath), devilish inclination (desire to do evil) and lordly inclination (desire to do good) (19). The personality is under the influence of the mind, and religion determines the direction of influence of mind. Religious principles give the mind the ability to distinguish right from wrong (akl-ı selim: le bon sens). Thanks to this ability, the mind prevents the desire to do evil and neutralizes the instincts of lusts/wrath, allowing the desire to do good to come to the fore in the personality. The opposite is also possible. In the presence of a mind that prefers to follow the evil, wrath and desire impulses instead of taking precautions for and blocking to them, the heart may become open and ready to do evil (21). While the desire to do good mediates the positive reinforcement of a behavior, the elements of desire to do evil, wrath or lusts can play the role of the negative reinforcement (see **Figure 4**).

The Islamic literature emphasizes the reason that the man’s very existence in the world is to exhibit the best behavior. As the Qur’anic verse says that “He who has created death as well as life, so that He might put you to a test [and thus show] which of you is best in conduct (Al-Mulk, The Dominion, 67/2) (23). Some Islamic provisions impose some actions/inactions shaping the heart, mind and self-devices which all are effective in the person’s behavior. The aim of these obligations is, actually, to protect them; with daily prayer (for heart), with periodic fasting (for self), and an indefinite ban on substance usage (for mind). Prayer (salah), which is expressed in the Quran (Al-Ankebut, The Spider, 29/45) as “An action that prevents a person from evil” differs from all other religious provisions in that it covers the childhood (24). As the Last Prophet Hz. Muhammad (may Allah’s blessings and peace be upon him) pointed out that “Teach your children to pray when they are 7, and lead them to pray when they are 10 years old” (25). It is particularly noteworthy that this commandment was uttered for an age group, that had not yet entered adolescence which was not at all binding in terms of religious obligation.

Prayer (salah), which is a set of actions consisting of repetitive motor movements and repeated reading of the Quranic expressions, is performed at certain times of the day and five times during the day. The developmental feature of the cognitive structure may lie behind the fact that the act of prayer should be taught at the age of 7. The age range of 7-11, which corresponds to the concrete-operational period of the cognitive development, is the age when the logical thinking skills begin to develop in the child. By the age of 7, the child begins to perform inductive logic, that is, reasoning from certain/specific information to a general principle on concrete objects and events (26). In this period, where playing games is replaced by a structured formal education, the ability to distinguish (notice) good from bad, which the Ghazali refers to as the light of the mind (nur-ı akl) takes shape (27). On the other hand, the age of 10 mentioned in the hadith is the age as is just before puberty when many physical changes at the biochemical level begin to emerge in the child (all physiological markers playing a role in growth and development including sex hormones) (28). It is undeniable that in this period before the transition to adolescence, as repetitive motor movements will contribute to the child’s physical development, as the repetitive Qur’anic expressions could very well affect to his/her mental functions. In fact, a recent study showed that reciting the Qur’an have posivite effect on mental health (29). Another study, carried out by Moulaei et al. (2023) showed that Qur’anic recitation have reducing effect on anxiety, stress and depression (30). There is still none any longitudinal study pointing out the Qur’anic repetition in a prayer could have effect on childhood group, nonetheless it is hypothetically valid and worth examining. The contributions of physical movements and repetitive routines (such as yoga and meditation) to the physical and mental health have been shown in many studies. Activity- and occupation-based approaches like meditation, animal-assisted interventions, creative arts, sports and yoga result in an improvement in the children and adolescents’ well-being, positive behavioral patterns, social participation, and mental health (31). The practice of prayer that will be carried out on a daily basis with repetitive bodily postures and physical movements, can contribute to the health, positive behavior and social participation of the child/adolescent just like meditation and yoga can. Indeed, as an activity of daily living (ADL), prayer has been shown to have a positive effect on physical health parameters (32).

It is well known that repetitive movements develop motor programs (skills) by being processed on procedural memory, which is a form of long-term memory (33). In this context, the repetitive Qur’anic expressions during “prayer” may very well prepare the ground for religion-oriented cognition through procedural memory. Based on a religious cognition, thanks to the inductive logic and reasoning (going from the part to the whole), a moral foundation begins to build in children. Thanks to this foundation, the child will be able to use her/his religious cognition as a reference during the transition to adolescence. The answer to the existential questions related to identity, purpose and meaning that dominate the period in adolescence may itself be religious cognition. In this case, the course of identity, self and autonomy development and the related behavioral preferences may be shaped on the basis of religious cognition (34). These preferences may contain a quality that will prevent many behavioral problems that are likely to arise during adolescence (school refusal, substance use, harming oneself or others, committing crimes, examples can be multiplied) before even they develop. Regardless of the biopsychosocial factors that the adolescent has, it is obvious that a behavior can be shaped according to the existence of religious identity developed. Since the characteristic of choosing good/right behavior, which dominates the religious personality, predicts the behaviors that the person will exhibit. Empirically demonstrated results support this hypothesis. A review study reported that the act of prayer strengthens mental health by reducing anxiety, depression, and anti-social tendencies (35). Another study has shown that praying is a strong coping mechanism and is associated with healthy behavior in children aged 8-12. In this study, Rew et al. (2004) investigated the possible effects of praying or not praying when they were stressed, tense or anxious on 150 girls and 121 boys with an average age of 10.4 (8-12 years). It was found that praying was positively related to social connectedness and sense of humor, and children who prayed frequently exhibited significantly higher levels of positive health behaviors than children who never prayed (36).

The development of a religious identity that will be shaped in adolescence seems possible by starting to perform prayer at the age of 7 to 10. In this context, the key in preventing some behavioral problems in adolescence and later adulthood is in the hands of parents or caregivers. By incorporating the act of prayer into the daily routines of their children aged from 7 to 10, parents or caregivers can prevent some behavioral problems that come to the fore during adolescence before even they develop.

From Islamic point of view, as the brain is only an interface between the heart and reseptor/effector, and the heart is the central decision-maker for a behavior, the religion’s attitudes to the human behavior differentiate from medical approaches. Given the inability to solve some problematic behaviors with current medical interventions, the lack of consideration of the person’s spirit device might be responsible for this.

In conclusion, the case formulation approach of the medical sciences, especially psychiatry, through the biopsychosocial model does not always yield satisfactory results in some problematic behaviors. Interventions based on the biological and psychological elements of the individual to resolve a problematic behavior are medications and psychotherapies. These interventions are effective via the brain-mind pathways. Yet, seeing the psychiatric symptoms in one-way axis (like body-mind) and intervening to it does not in yield adequate results, provided the fact that human being is a biopsychosocial entity. The fact that a problematic behavior sometimes cannot be solved despite these interventions can be explained by ignoring the fact of religion, one of the social factors affecting the exhibition of the behavior. According to the Islamic literature, human knows his/her Creator thanks to the soul device in his/her body and turns to Him via the heart device. Thanks to the religious practices, especially prayer, a person who exhibits behavior on the mind-heart axis reaches a cognitive, emotional and behavioral norm with the religious personality she/he develops. The practice of prayer that will be carried out on a daily basis with repetitive bodily postures and Quranic expressions, can contribute to the health, positive behavior and social participation of the child/adolescent just like meditation and yoga can. In this perspective, integrating the act of prayer into the daily routines of the children aged 7 to 10 could only be possible via their parents’ guiding. Daily prayer routine, thus, can take its place among the activities of daily living (ADLs) of childhood, and stands out as an element that will be useful in eliminating many problematic behaviors that are likely to develop during adolescence before even they develop.

## Data availability statement

The original contributions presented in the study are included in the article/supplementary material. Further inquiries can be directed to the corresponding author.

## Author contributions

ZG: Conceptualization, Investigation, Methodology, Software, Visualization, Writing – original draft, Writing – review & editing.
